# Palliative care integration: a critical review of nurse migration effect in Jamaica

**DOI:** 10.1186/s12904-021-00863-7

**Published:** 2021-10-13

**Authors:** Rebecca L. Edwards, Patricia A. Patrician, Marie Bakitas, Adelais Markaki

**Affiliations:** 1 Department of Acute, Chronic, and Continuing Care, School of Nursing, University of Alabama at Birmingham, 1720 2nd Avenue South, Birmingham, AL 35294-1210 USA; 2 Family, Community and Health Systems Department, School of Nursing, University of Alabama at Birmingham, 1720 2nd Avenue South, Birmingham, AL 35294-1210 USA; 3 Center for Palliative and Supportive Care, School of Nursing, University of Alabama at Birmingham, 1720 2nd Avenue, South, Birmingham, AL 35294-1210 USA; 4 PAHO/WHOCC for International Nursing, Family, Community and Health Systems Department, School of Nursing, University of Alabama at Birmingham, 1720 2nd Avenue South, Birmingham, AL 35294-1210 USA

**Keywords:** Palliative care, Integration, Nurse migration, Jamaica, CARICOM, Caribbean, Critical review

## Abstract

**Background:**

Provision of palliative care to individuals with late-stage serious illnesses is critical to reduce suffering. Palliative care is slowly gaining momentum in Jamaica but requires a highly skilled workforce, including nurses. Out-migration of nurses to wealthier countries negatively impacts the delivery of health care services and may impede palliative care capacity-building. This critical review aimed to explore the evidence pertaining to the nurse migration effect on the integration of palliative care services in Jamaica and to formulate hypotheses about potential mitigating strategies.

**Methods:**

A comprehensive search in the PubMed, CINAHL, and ProQuest PAIS databases aimed to identify articles pertinent to nurse migration in the Caribbean context. Grant and Booth’s methodologic framework for critical reviews was used to evaluate the literature. This methodology uses a narrative, chronologic synthesis and was guided by the World Health Organization (WHO) Public Health Model and the Model of Sustainability in Global Nursing.

**Results:**

Data from 14 articles were extracted and mapped. Poorer patient outcomes were in part attributed to the out-migration of the most skilled nurses. ‘Push-factors’ such as aggressive *recruitment* by wealthier countries, lack of continuing *educational* opportunities, disparate wages, and a lack of professional autonomy and respect were clear contributors. Gender inequalities negatively impacted females and children left behind. Poor working conditions were not necessarily a primary reason for nurse migration. Four main themes were identified across articles: (a) *globalization* creating opportunities for migration, (b) *recruitment* of skilled professionals from CARICOM by high income countries, (c) *imbalance and inequities* resulting from migration, and (d) *mitigation strategies*. Thirteen articles suggested *education, partnerships, policy,* and *incentives* as mitigation strategies. Those strategies directly align with the *WHO Public Health Model* drivers to palliative care integration.

**Conclusion:**

Emerged evidence supports that nurse migration is an ongoing phenomenon that strains health systems in Caribbean Community and Common Market (CARICOM) countries, with Jamaica being deeply impacted. This critical review demonstrates the importance of strategically addressing nurse migration as part of palliative care integration efforts in Jamaica. Future studies should include targeted migration mitigation interventions and should be guided by the three working hypotheses derived from this review.

## Background

Palliative care is a team-based holistic approach that focuses on improving quality of life and reducing suffering in those with serious illnesses [1, 2]. It is well-established that palliative care should be integrated at the time of diagnosis or early in the serious illness trajectory [3–8]. The United Nations (UN) has declared palliative care a human right [9] and the World Health Organization (WHO) has called for governments to integrate *primary* palliative care in the continuum of care, as part of universal health coverage schemes [10]. In the United States of America (USA), palliative care is available through specialized teams in 72% of larger hospitals [11]. In contrast, palliative care in Jamaica is provided by only two specialists [10] at hospital-based clinics in three out of 24 public hospitals. Community-based palliative care is largely unavailable, with only a few privately funded small initiatives, and nearly all palliative care recipients have cancer diagnoses [12, 13]. Thus, limited palliative care availability in Jamaica leads to a significant disparity for patients with serious illnesses. An adequately trained and available healthcare workforce is necessary to ensure access to quality palliative care. However, the *migration* of health workers, and particularly of nurses, stands out as a potential barrier. Understanding the phenomenon of nurse migration and ensuring that adequately skilled nurses are retained is of utmost importance in palliative care integration efforts, especially in light of the ongoing COVID-19 pandemic.

Health personnel migration takes place in the context of larger migration trends [14] and leads to inadequate health system performance in developing countries [15]. Typical migratory patterns involve health personnel leaving developing countries, such as Jamaica, and relocating in wealthier countries such as the USA, the United Kingdom, and Canada, resulting in an overall inequitable and unsustainable balance [16]. Shortages secondary to migration and other factors constitute a major threat to less developed health systems and to global health security [17], undermining efforts to meet the UNs’ Sustainable Development Goals (SDGs) [18]. In 2010, the *WHO Global Code of Practice on International Recruitment of Health Personnel* [19] was adopted to serve as a framework for global dialogue and cooperation, mainly around recruitment from developing countries facing critical shortages of health personnel [19]. The Code calls for more ethical recruitment, avoidance of recruitment from countries with skilled health worker shortages, and careful monitoring of international mobility of health workers [14].

Nurse migration has been viewed as a potential threat to global health security [7]. Although nurses make up more than half (59%) of the world’s healthcare workforce, accounting for 27.9 million, the overall shortage of nurses was estimated at 5.9 million in 2018 [20, 21]. The importance of nurses in achieving the SDGs and universal health is underscored by the *State of the World’s Nursing 2020 Report* [21]. Severe nursing shortages in developing countries, compounded by aging populations, increased health service specialization, and technological complexity are major contributors to nurse migration [22]. In the Caribbean, nurses migrate for many reasons including better remuneration, improved work conditions, and career advancement [23, 24]. Historically, nurse migration has resulted in an imbalance of the skilled nursing workforce favoring wealthier countries; a phenomenon expected to continue in Jamaica and the wider Caribbean without significant intervention [16].

The Caribbean Community and Common Market (CARICOM) is a group of 20 developing countries in the Caribbean that have united to improve standards of living and work and to enhance levels of international competitiveness [25]. A scoping review by Rolle Sands et al., which was published after our search was completed, examined the conceptual and empirical literature pertaining to nurse out-migration from the CARICOM region [24]. Their findings supported a need for future research on the impact of nurse migration on health systems and population health within Caribbean countries. Furthermore, the need to address palliative care educational deficits in the Caribbean and, more specifically, in Jamaica has been underscored [13]. Jamaica, an English-speaking country with a population of 2.8 million, is a CARICOM member state that has been profoundly affected by nurse migration [26]. Jamaica has the highest emigration rate in the CARICOM; 745,000 (16.9%) in 2017, with skilled professionals consistently constituting a high share of those who emigrate [27]. In fact, 85% of Jamaica’s skilled labourors emigrated to wealthier countries from 1965 to 2000; twelve times higher than emigration rates of wealthier countries and eight times higher than the world average [28]. Jamaica’s nursing shortage is a product of longstanding nursing shortages in wealthier countries [29, 30] coupled by increasing global demand due to aging and patient care complexities [31]. Nurse burnout related to high patient care stress in wealthier countries exacerbates the demand for Jamaican nurses, as does an overall shortage of nurse faculty, which causes qualified student applicants to be turned away from nursing school admission [32]. Jamaican nurses are attractive recruits because they are English-speaking, have high pass rates on licensure exams, and are highly skilled [33]. These nurses are attracted to wealthier countries by the promise of higher salaries. The starting salary in 2015 for a nurse in Jamaica was $8000 compared to $65,000 in the USA [34]. Although nurse migration statistics specific to Jamaica are not readily available, in 2018, the density of nurses per 10,000 population was 8.07 in contrast to 13.36 in the USA [35]. By 2025, the projected unmet demand for nurses in the CARICOM region will be 10,700 [36]. The Jamaican Minister of Health, Dr. Christopher Tufton, proclaimed, “*brain drain* [a slang term for migration] has virtually crippled the delivery of certain health care services and has had a dramatic effect on the overall quality of health care [in Jamaica] [17].”

Given the escalating nursing shortage, it is important to factor migration into palliative care expansion efforts in Jamaica. The country’s *Strategic Plan and Action Plan for the Prevention and Control of Cancer* offers evidence that palliative care is gaining momentum [37]. Nursing attributes align naturally with core fundamentals of palliative care, thus, nurses are ideally suited to lead efforts to integrate palliative care, as long as they can be trained and retained [38]. To effectively integrate palliative care in a country’s health system, the *WHO Public Health Model* (PHM) calls for policies, availability of essential medicines, and comprehensive education [39, 40]. These components are necessary to ensure that palliative care is embedded in existing health systems and that specialist services are available for the most complex patients. The *Sustainability in Global Nursing Model* supports that empowering nurses leads to sustainably strengthening the nursing workforce [41].

Guided by the above two models, we examined nurse availability as a facilitator and nurse migration as a barrier to palliative care integration in Jamaica. Our intent was to better understand ways to mitigate ‘push-factors’; factors influencing emigration of health professionals from their home country. The Rolle-Sands et al. [24] review identified sustainable partnerships across Jamaica and wealthier countries as important to facilitating autonomy and advancing nursing practice in the CARICOM. These findings align with our academic partnership experiences pursued through two Pan-American Health Organization(PAHO)/WHO Collaborating Centers (WHOCCs); one based at the University of Alabama at Birmingham (UAB) in the USA, and another at the University of West Indies (UWI) in Jamaica. This academic partnership between WHOCCs has led to research immersion, simulation training, visiting scholar exchanges, and development of distance-accessible courses [42, 43]. Hence, the purpose of this critical review was to explore the evidence pertaining to the nurse migration effect on the integration of palliative care services in Caribbean countries with a special focus on Jamaica, and to formulate hypotheses about potential mitigating strategies. Our guiding questions were: (a) What is the effect of nurse migration on palliative care in Caribbean countries and, specifically, in Jamaica?, (b) What are the potential nurse migration-related barriers to palliative care integration in Jamaica?, and (c) Which nurse migration mitigation strategies should be recommended to optimize palliative care integration in Jamaica?

## Methods

We utilized Grant and Booth’s methodologic framework for critical reviews to evaluate the literature following a narrative, chronological synthesis with the goal of developing working hypotheses [44]. This methodology involves: (a) appraisal focused on the article’s overall contribution to knowledge; (b) synthesis using a conceptual narrative to identify themes across articles; and (c) analysis of these themes in relation to the WHO PHM [39, 40] and the Sustainability in Global Nursing Model [41].

### Search strategy

**Fig. 1:**
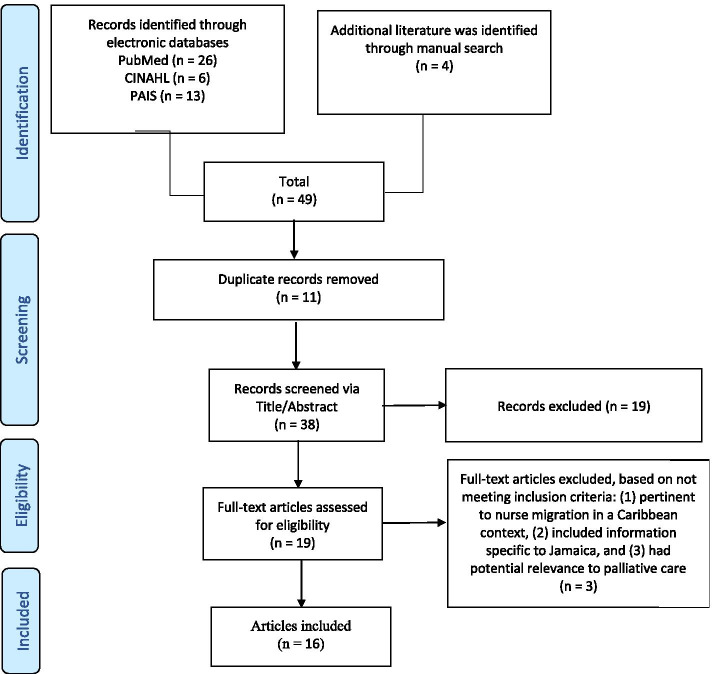
PRISMA Literature Search Diagram [45]

The PubMed, CINAHL, and ProQuest PAIS databases were searched to capture biomedical, nursing and allied health, and public health literature. Filters applied were: 1) peer reviewed articles, 2) available in English language, and 3) published between 2004 and July 2019. We sought to identify articles meeting the inclusion criteria: (1) pertinent to nurse migration in a Caribbean context, (2) included information specific to Jamaica, and (3) had potential relevance to palliative care. Search terms across databases included “*nurse AND migration AND Jamaica”* and “*nurse AND migration AND Caribbean*”. The terms “*nurse AND migration AND Jamaica AND Palliative*” and “*nurse AND migration AND Caribbean AND palliative*” did not result in additional relevant articles. The query resulted in 45 articles, with an additional four hand-searched articles added. After removing duplicates, titles and abstracts were screened for relevance, leaving 19 articles for full-text review. These 19 articles were reviewed for inclusion criteria and three articles were eliminated. This left 16 articles that comprised the final yield as displayed in the PRISMA diagram (Fig. 1) [45].

### Data extraction and analysis

Included articles were mapped by origin, type of article, level of evidence, purpose, and key points. This mapping allowed for comparisons and resulted in determination of themes most pertinent to nurse migration in the CARICOM. Appraisal of level of evidence was carried out according to the adapted *Rating System for the Hierarchy of Evidence* [46, 47].

### Findings

**Fig. 2:**
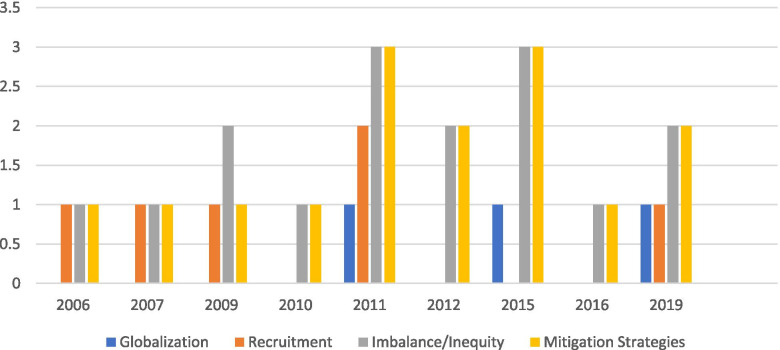
Themes by Publication Year

Extracted data from the 16 sampled articles were entered chronologically in Table 1 and emerged themes were determined. Although no articles specifically addressed nurse migration in relation to palliative care, we extracted and synthesized evidence useful in understanding how nurse migration might affect optimal palliative care integration in Jamaica. Only five articles were appraised at level VI in the hierarchy of evidence and included a qualitative case study [48], a qualitative study using focus groups [49], a quantitative exploratory descriptive study [50], a quantitative descriptive correlational study [51], and a mixed methods study [52]. The remaining articles included a literature review (level VII) [53], a World Bank report [54] and an International Organization for Migration (IOM)/UN report (Level VIII) [55], and eight commentaries [29, 30, 32, 33, 56–59]. Four main themes were identified across articles: (a) *globalization* creating opportunities for migration, (b) *recruitment* of skilled professionals from CARICOM by high income countries, (c) *imbalance and inequities* resulting from migration, and (d) *mitigation reduction strategies*. The distribution of themes by publication year is depicted in Fig. 2.

**Table 1:** Data extraction and appraisal of selected articles in chronologic order (*n* = 16)

Author(s), Year [Ref#]	Region or Country	Purpose	Type of Article/Level of Evidence	Key Points	Emerged Themes (sub-themes)
Yan, 2006 [29]	Caribbean	To describe the CARICOM MMP as an intervention to reduce negative repercussions of nurse migration.	Commentary/Level VIII	1. High income countries aggressively recruit from CARICOM countries. 2. Nursing shortages in CARICOM countries lead to negative impacts in healthcare quality, loss of money, and undesirable working conditions for remaining nurses. 3. MMP guiding principles; observe nurses’ rights while balancing obligations to provide high quality healthcare. 4. MMP critical areas; (1) terms and conditions of work, (2) recruitment, retention, and training, (3) value of nursing, (4) utilization and deployment, (5) management practices, and (6) policy development. 5. Jamaica/Florida partnership; 2 weeks alternating in each country. 6. Brain gain: Caribbean nurses return home to share knowledge and skills. 7. Health and tourism; bring nurses from wealthy countries to tourist destinations in the Caribbean for 6 mo. to 1 yr.; pay is same as for local nurses.	- Recruitment - Imbalance/inequity - Mitigation strategies (partnership, policy)
Salmon et al. (2007) [30]	Caribbean	To describe the nursing workforce issues in the Caribbean and the MMP as a framework for addressing nurse migration.	Commentary	1. Out-migration of nurses from the Caribbean is due to many (p. 1357). 2. The Caribbean feels the loss of highly trained nurses and nurse educators. 3. Jamaica has a critical shortage of nurses (58% vacancy rate in 2003) that compromises the quality of care. Some of the void This void is partially filled by recruiting from Cuba, Guyana, India, Ghana, Burma, Russia, and Nigeria. 4. Regional and international nurse collaborations offer some hope in addressing nurse migration (p. 1361). 5. The most significant regional achievement is the MMP. 6. Factors supporting and detracting from MMP success are outlined (p. 1367). 7. GATS as a framework for nurse migration includes incentives for nurse to stay in-country. 8. Align MMP with SDGs and MMP must be in CARICOM agenda.	- Recruitment - Imbalance/inequity - Mitigation strategies (partnership/policy)
Jones et al. (2009) [53]	Caribbean	To explore the significance of gender in nurse migration from the Caribbean to the U.K.	Literature review/Level VII	1. Gender issues are significant in all aspects of the migratory process. 2. Migrant nurses contribute to social progress in the Caribbean on their return, but an overall negative impact exists, particularly for women. 3. Women experience gender inequalities driving migration and gain economic freedom. 4. Gender inequalities persist in some higher economic environments upon migration. 5. ‘Feminization of migration.” 6. Women left behind pick up care responsibilities for family members, sick, elderly. 7. Female migrants are more reliable remitters than men. 8. Migration leads to family separation and emotional costs.	- Imbalance/inequity
World Bank (2009) [54]	English-Speaking Caribbean	To produce a comprehensive assessment of the nurse labor and education markets of the English-Speaking CARICOM	Expert Opinion/Level VIII	1. Nurses are a globally scarce human resource 2. Data limitations are a significant issue 3. < 10% of nurses in primary care 4. > 90% of nurses employed in the public sector 5. Nurse to population ratios ten times lower than in wealthier regions 6. Demand for nurses exceeds supply 7. 8% annual attrition mainly due to outmigration 8. The brightest nurses out-migrate driven by wage differentials, network effects, and worker dissatisfaction 9. Nursing schools are public, semi-autonomous, and private 10. Trend towards students paying for their own tuition 11. Approximately 50% of students never complete their program – concerns for poor quality of education 12. Insufficient nurse tutors 13. Average of three qualified candidates competing for every position in nurse training programs 14. Each CARICOM country has improved the quality of their programs (curricula, pedagogical approaches, harmony across programs) 15. Estimated gap between demand for and supply of nurses will widen from 3400 (2009) to 10,700 in 2025 16. Strategy to mitigate nurse shortages = expand training capacity, maximize program completion rates 17. Financing nurse training through students, local, and foreign governments 18. Policy must address training completion rates, training capacity, managed migration, mobilizing the inactive supply of nurses	- Recruitment - Imbalance/inequity - Mitigation strategies (partnership, policy, education)
Senior (2010) [32]	Caribbean	To highlight the shortage of nurses in developing countries for International Nurses Day, 2010.	Commentary	1. The Caribbean will have a nursing shortage of 10,000 by 2025. 2. Push factors to leave the Caribbean, increased pay, better conditions, professional incentives. 3. Nurse migration leads to poorer quality healthcare and worse health indicators (e.g., infant mortality). 4. Paucity of educational opportunities is a major factor in nurse migration. 5. Loss of nurse faculty; retirement. Nurse to faculty ratios in Caribbean 45:1 and in wealthy countries 12:1. 6. ICN, International Education Network in collaboration with the National League for Nurses; a collaboration to address nurse shortages. 7. WHO global standards for professional nurse education. 8. Bologna Process allowing academic exchange in Europe.	- Imbalance/inequity - Mitigation strategies (policy, education)
Adelberger et al. (2011) [50]	Bahamas	To collect nurse migration data from the Bahamas from 1994 to 2005.	Exploratory descriptive/Level VI	1. Nurse migration in the Bahamas is lower than in the surrounding Caribbean; stronger economy, better pay, a robust public health core contribute.	- Imbalance/inequity - Mitigation strategies (policy)
Evans and Tulaney (2011) [56]	Jamaica and Philippines	To examine the impact of nurse migration.	Commentary	1. To understand migration, one must first understand globalization. 2. Geographic maldistribution of nurses; barriers to care for rural dwellers. 3. Recruitment from the U.S.A. is problematic. 4. Push factors are related to education and practice. 5. Estimating the severity of nurse migration in Jamaica is challenged by poor registration data. 6. Philippine nurses encouraged to migrate and remit, Jamaican nurses leave related to push-pull factors. 7. Strategies like MMP are needed as is scientific investigation.	- Globalization - Recruitment - Imbalance/inequity - Mitigation strategies (partnership/policy)
Lewis (2011) [33]	Jamaica	To discuss training nurses for export and the implications of this practice, using Jamaica as an example,	Commentary	1. Nurse and educator migration from Jamaica accounts for 70% of nurse attrition. 2. Remittances account for 12% of the gross domestic product. 3. Shift from brain drain to brain circulation and diaspora; focus on positives of migration (e.g., economic, skill, lower fertility, lower unemployment, etc.) 4. Negative implications of migration are significant (e.g., split families especially in poor households). 5. Nursing crisis: push factors outweigh pull factors. 6. Lack of professional autonomy and authority; pay not tied to level of education and experience so nurses demotivated to stay. 7. Aging of nurses; leaving workforce. 8. Training capacity in Jamaica is critical and well described in this article including costs and Ministry of Health and Wellness involvement. 9. Training for export; hemorrhaging of skilled people in trade for remittances and hope for return migration and skill. 10. Formal partnerships between U.S.A. and Jamaica. 11. National, regional, and international strategies - National; training for export, focus on education - Regional; MMP - International; Commonwealth Code of Practice for the International Recruitment of Health Workers, GATS	- Recruitment - Imbalance/inequity - Mitigation strategies (partnership, policy, education, incentives)
Lansiquot et al. (2012) [51]	Eastern Caribbean	To describe the relationship between practice environment and intention to leave in hospital-based nurses.	Descriptive correlation/Level VI	1. The practice environment was described as generally unfavorable but had little to do with nurse intention to leave. Most nurses were single, young, and had entry-level nursing education. There is a high intention to leave overall	- Imbalance/inequity - Mitigation strategies (education)
Lofters (2012) [59]	Jamaica	To describe “brain drain” in developing countries, highlighting Jamaica as a country that illustrates the complexity of the issue, and to discuss creative and sustainable solutions to the problem.	Commentary	1. The loss of health workers from developing countries has devastating impacts. 2. $500 million annually to train health workers who leave to higher income countries. 3. Moving within country to urban from rural areas is a predictor for overseas migration. 4. Active recruitment by wealthy countries, poor conditions in home countries. 5. ‘Brain drain perpetuates brain drain.’ 6. Solutions are not simple or clear. Global Forums on Human Resources, Global Health Workforce Alliance, 2008 and 2011. Goal to improve resources for health to reach SDGs. WHO Global Code of Practice on the International Recruitment of Health Personnel. 7. Wealthy countries need to take responsibility and not take advantage. 8. Jamaica as an example: It is hardest hit by migration. About 80% of all Jamaicans with higher education have migrated out. Nurse migration of 8% of registered nurses and 20% of specialists; two thirds have migrated. High vacancy rates for all nurses; overall 58% in 2003. 9. Nurse migration negatively impacts healthcare quality and outcomes, production, economy. With so many females leaving their families behind, “barrel” children do more poorly in school, have delinquent behaviors, and are more likely to be sexually abused. 10. Jamaica has resorted to recruiting nurses from other countries (Ghana, Nigeria, etc.). Cyclical, perpetual effect. 11. Jamaica and partners invested in ethical solutions; MMP, IOM national migration policy, etc.	- Imbalance/inequity - Mitigation strategies (partnership, policy)
Clark et al. (2015) [48]	Haiti	To highlight nursing continuation education as a strategy to retain nurses in developing countries providing an example of this in Haiti.	Case study/Level VI	1. Haiti and other developing countries suffer nurse shortages partly due to migration and lack of retention. 2. Nursing continuing education has the potential to positively impact nurse retention and to enhance nurse performance but is underutilized. 3. Eight dimensions of nursing continuing education for success. - involve key stakeholders - target to nurse participant level and area of care - base course content on local context - use diverse nursing topics - use participatory teaching techniques - address resource constraints in time and scheduling - evaluate and monitor outcomes - establish partnerships 4. This continuing education process is possible with commitment and engagement and is an important strategy in retaining nurses.	- Imbalance/inequity - Mitigation strategies (education, partnerships)
Johnston et al. (2015) [60]	Barbados	To determine perceptions of medical tourism; an expanding industry.	Qualitative/Level VI	1. Three core concerns about medical tourism. - incentivizes migration - burdens the tertiary healthcare system - produces different tiers of quality of care 2. These concerns are framed by: - both universal public and private medical insurance-based medical sectors - international mobility between health workers and patients - a large recreational tourism sector	- Imbalance/inequity - Mitigation strategies (incentives)
Jacobson (2015) [58]	Latin America and the English-speaking Caribbean	To discuss nurse migration	Commentary	1. Nursing shortages are severe. 2. Globalization of nursing; migration has benefits. 3. Nurses remit part of pay back home and boost economy. 4. Training and education are not uniform across countries. 5. Nurses do not leave for better salaries only. It has more to do with deficient work environments. 6. Nursing education is an investment that is key to retention, educational pathways and opportunities for advanced practice education. 7. Nurses also need to be integrated in policy decisions, but education must be prioritized.	- Globalization - Imbalance/inequity - Mitigation strategies (education, policy)
Tomblin Murphy et al. (2016) [52]	Jamaica	To determine drivers of health worker migration	Mixed methods/Level VI	1. Health worker migration from Jamaica is prevalent and due to differences in working and living conditions in Jamaica and ‘destination’ countries. 2. Formal tracking of HCW from Jamaica is lacking. 3. Outcomes from multiple efforts to reduce migration have not been well described. 4. Additional strategies that may be helpful: information systems to formally monitor migration, updating the national cadre system that tracks health worker employment, enforce existing personnel management strategies, use formal and informal health worker recognition.	- Imbalance/inequity - Mitigation strategies (partnership, policy, incentives)
George et al. (2019) [57]	Caribbean	To describe the wage gap and financial incentives leading to human resources for health (HRH) migration within and from the Caribbean.	Commentary	1. Medical doctors, registered nurses, and specialists are included in the analysis of salaries. 2. Nurses who migrate to destination countries have substantially higher earning power (214.2% more in the U.S.A.). 3. When strategizing to reduce nurse migration, governments have to consider earning potential in destination countries.	- Imbalance/inequity - Mitigation strategies (incentives)
International Organization for Migration – UN (2019) [55]	Jamaica	To provide a descriptive analysis of the main migration characteristics and trends in Jamaica from 2011 to 2015	Expert Opinion/Level VIII	1. Globalization has had significant negative impacts on Jamaica’s economy. 2. 2256 registered nurses in Jamaica (2005), 58.3% vacancy rate (2009). 3. Nurses recruited to the USA and Canada through private institutions. 4. Gaps in specialized nursing – 74% in public health, 70% in nurse anesthesia, 68% in psychiatry, 106% in critical care, 111% in pediatrics, 113% in emergency settings, 32% in oncology… 5. Overall, 54% nursing shortage – ‘depletion of nurses in some specializations,’ 6. ‘Bonding’ as a strategy to keep nurses in Jamaica but nurses just pay off their bonds and out-migrate 7. Increase training output to bolster nurse retention	- Globalization - Recruitment - Imbalance/inequity - Mitigation strategies (partnership, policy, education, incentives)

### Globalization

The interconnectedness of countries by flow of goods, services, money, people, information, and ideas [61] was highlighted as a key reason for nurse migration [56, 58]. Evans and Tulaney suggested that one must first understand globalization to understand nurse migration [56]. Jacobson added that globalization affects a nurse’s decision to migrate based on the benefits associated with this practice (e.g. higher pay and improved workplace conditions) [58]. Higher salaries and the ability to send remittances (i.e., a portion of salary) home are factors in a nurse’s decision to migrate. However, other considerations are ‘push’ factors that lead to out-migration. For instance, high patient-nurse ratios and lacking the resources to adequately care for patients and families. Educational opportunities are few and governments under-invest in nursing education, limiting the ability of predominantly female nurses to advance in the field [58]. Although not specific to palliative care, globalization can potentially negatively impact palliative care integration in countries with low resources.

### Recruitment

Nurse *recruitment* was highlighted in six articles as a main contributor to nurse migration [29, 30, 33, 54, 56]. Aggressive *recruitment* by higher income countries leads to a maldistribution of nurses across regions. Nurses with higher education and skill move to wealthier countries leaving more novice nurses in the CARICOM and resulting in nurse shortages, poorer working conditions, and barriers to optimal care. A World Bank report discussed instituting regional recruitment policies in the CARICOM to thwart active recruitment by wealthier countries [54]. The IOM-UN report suggested that some recruitment for tertiary education and employment is through unregistered agencies, and that the welfare of recruits is uncertain, which argues for greater surveillance of recruitment practices [55]. Three articles called attention to training for export; deliberate government policies that focus on training professionals from developing countries to fill positions in wealthier countries that are in short supply [29, 33, 54]. This practice leads to an exodus of skilled people from the CARICOM countries in exchange for salary remittances that boost the economy, and a false hope that these individuals will return home with added skills. Training for export models are heavily utilized in the Philippines but are supported through private funding, which is not readily available in Jamaica. Exporting nurses also leads to ongoing nurse shortages and an exaggerated need for nurse education and training opportunities that are already lacking. Although not specific to palliative care, recruitment of nurses by wealthier countries and training nurses for export could negatively impact palliative care integration in low resource countries.

### Imbalance and inequalities

All 16 articles highlighted the profound *imbalance and inequities* caused by nurse migration from the CARICOM, with eight articles highlighting Jamaica [29, 30, 33, 52, 54–56, 59]. Generally, *imbalance and inequities* had to do with nursing shortages, loss of specialists and highly skilled nurses, economic and social impacts, and the resulting poor work conditions in the CARICOM countries. Gender was a key issue related to nurse migration in two articles [53, 59]. Historically, women migrated to wealthier countries to reunite with their spouses or other family members. However, labor shortages in these countries have led to a ‘feminization of migration’ with skilled women representing an increasing proportion of the global migrant workforce [53]. Unfortunately, when women leave a country, the remaining female family members become burdened with caregiver responsibilities. Further, gender inequalities in the home countries often continue for skilled migrant women in the receiving countries [53]. Many women who out-migrate leave their children behind, and although they may send financial support for child care, there is an emotional cost to this family separation [53]. The ‘barrel children’ left behind do more poorly in school, have more delinquent behaviors, and are more likely to be sexually abused [59]. Hence, health system imbalances and inequalities could potentially negatively impact palliative care integration in countries with very limited resources.

### Mitigation strategies

The CARICOM’s Managed Migration Program (MMP) highlighted key strategies for recruitment, retention, and training of nurses, terms and conditions of work, valuing the nursing profession, and managed nursing practice [29, 30, 33, 56, 59]. Fifteen articles suggested mitigation strategies which were grouped into four categories: *education, partnerships, policy,* and *incentives*.

Seven articles featured nursing *education* as a strategy to retain nurses noting the paucity of educational opportunities as a major factor in migration [32, 33, 48, 51, 54, 55, 58]. For instance, Lewis [33] suggested that training capacity for nurses in Jamaica was insufficient and that nurses were demotivated to stay. Clark et al. [48] highlighted continuing education strategies to positively impact nurse retention. Jacobson [58] suggested that investing in nursing education was key to nurse retention and may include educational pathways and opportunities for advanced practice education. The World Bank report heavily focused on nurse training as a key component to mitigate out-migration of nurses [54]. And the IOM-UN report discussed increasing nurse training output to improve nurse vacancy rates, particularly in specialty areas such as critical care and public health [55].

Eight articles identified *partnership*s as a key strateg*y* in addressing migration [29, 30, 33, 48, 52, 54, 56, 59]. For instance, Yan [29] highlighted the MMP’s Jamaica-Florida partnership where nurses alternate between the two regions resulting in ‘brain gain’, or a homecoming of sorts where nurses who have migrated to Florida return to Jamaica and share newly acquired skills and knowledge. Clark et al. [48] discussed partnerships to bolster continuing education opportunities in developing countries, while Lewis and the World Bank report [33, 54] suggested formal USA-Jamaica partnerships to mitigate nurse migration. The World Bank report highlighted a need for Caribbean countries to partner with wealthier countries to increase nurse tutoring capacity, because this is a key deficit in Caribbean nurse training programs [54]. However, the IOM-UN report suggested that Jamaica’s partnering through temporary migration with the USA and Canada should continue with caution due to competitiveness with domestic labor that may result in altered and substandard agreements [55].

*Policy-related strategies* were a focus in 11 articles [29, 30, 32, 33, 50, 52, 54–56, 58, 59]. For example, Tomblin Murphy et al. [52] discussed instituting information systems to formally monitor migration and updating the national cadre system to track health worker employment. Salmon [30] highlighted the General Agreement on Trade and Services (GATS) as a framework for nurse migration. One element of the GATS framework calls for the *temporary* movement of labor to supply services overseas with return of skilled workers with new knowledge and experiences as a replacement for permanent migration of professionals. This calls for harmonization across countries to ensure that professionals return home after a suitable stay [30]. Lofters [59] discussed policy initiatives that target nurse migration including Global Forums on Human Resources, the Global Health Workforce Alliance, and the WHO Global Code, pointing out that solutions to migration are not ‘simple or clear.’ Jacobson [58] highlighted that nurses must be integrated in policy decisions to effectively mitigate migration. The World Bank report [54] underscored multiple policy-driven mitigation strategies such as creating barriers to migration and establishing reintegration programs for returning nurses, recruitment codes of practice, mobilizing the inactive supply of nurses, allocating nurses to primary care settings, task-shifting to lower skilled workers, and facilitating private sector investment in nurse training. And the IOM-UN report highlighted ‘bonding’ as a practice to retain nurses who owe up to 5 years of service as repayment for government-funded education. However, this strategy often fails as nurses simply repay their government bonds and have high potential to out-migrate before the terms of their bond are fulfilled [55].

*Incentive-based strategies* were discussed in five articles [33, 49, 52, 55, 57]. George et al. [57] focused on the extreme wage gaps between CARICOM countries and ‘destination’ countries (e.g. nurses make 214% more in the USA), noting that CARICOM governments must consider earning potential in efforts to retain nurses. Lewis [33] offered that nurses are not motivated to stay in CARICOM nations due to lack of professional autonomy and authority, and that efforts to tie pay and practice privileges to education level and practice experience may be advantageous in mitigating nurse migration. Conversely, Johnston et al. [49] shed light on ‘medical tourism’ producing an incentive for Barbadian nurses to migrate from public to private medical facilities. Medical tourism is an economic development strategy to attract foreign patients by marketing affordable and accessible medical care [62]. This influx of privately paying tourists draws nurses to the private sector for higher paying jobs and results in shortages and inequitable care for local patients seen in the public health sector [62]. And the IOM-UN report discussed Jamaica’s *Return of Talent Program*, which offers financial incentives for return-migration for specific sectors and occupational groups. This report also highlighted other incentives for return-migration such as supplying job information, linkages with employers, and investment opportunities [55]. Although not specific to palliative care, these mitigation strategies are important to bolstering palliative care integration in countries with low resources.

## Discussion

This critical review was driven by the need to understand how nurse migration might affect palliative care integration in Jamaica. Emerged evidence supports that nurse migration is an ongoing phenomenon that strains health systems in CARICOM countries, with Jamaica being the most deeply impacted. Migration rates from some Caribbean countries, such as the Bahamas, are lower for reasons that are not clear but are likely related to wealthier economies and fewer workplace challenges [50]. The *inequities and imbalances* resulting from nurse migration were described across articles. Negative implications, such as poorer patient outcomes (e.g., infant mortality), were in part attributed to the out-migration of the most skilled nurses. ‘Push-factors’ such as aggressive *recruitment* by wealthier countries, lack of continuing *educational* opportunities, disparate wages, and a lack of professional autonomy and respect were clear contributors. Poor working conditions (e.g. high patient-nurse ratios) may also contribute, but were not necessarily a primary reason for nurse migration [51], although worker dissatisfaction arose as a determinant for out-migration in the World Bank report [54]. Gender inequalities were also implicated as a push-factor for nurse out-migration, negatively impacting females and children left behind [53, 59]. A multitude of *mitigation strategies* to thwart nurse migration were highlighted including *education*, *partnership*, *policies*, and *incentives*. Providing educational opportunities and capitalizing on policy initiatives, such as the MMP, stood out as essential strategies to retain Caribbean nurses [48]. However, outcomes were poorly described and it was uncertain if these strategies were sufficient [52]. The World Bank report highlighted several policy initiatives that have potential to mitigate the negative impacts of nurse migration such as reintegration policies and recruitment codes [54]. And the IOM-UN report focused on ‘bonding’ to keep nurses in Jamaica based on obligations to fulfill up to 5-year positions in return for educational funding, however bond prices may need to be increased to prevent nurses from paying them off and out-migrating [55]. The IOM-UN report framed globalization based on early negative impacts particularly to the agriculture sector leading to overall economic decline, which pushed Jamaicans (including nurses) toward out-migration to the USA and Canada [55]. In a mixed methods dissertation study, although not included in the review based on not meeting inclusion criteria, Russell suggested that the Caribbean has participated in the creation of globalization due to an openness to migration and trade, leading to an internationalization of labor [63]. Russell also discussed the role of well-educated second generation migrants returning to their home countries to bolster social and economic factors [63].

### Nurse migration as a facilitator and barrier to palliative care

The emerged themes of *education*, *partnership*, *policy*, and *incentive-based mitigation strategies* directly align with the *WHO PHM* drivers to palliative care integration. These findings suggest that Jamaican nurses will be less apt to migrate with improved *educational* opportunities and revision of *policies* to include *incentives* for advanced *education* and leadership roles.

Expanding *educational* opportunities, such as interprofessional palliative care training would allow nurses to work alongside other professionals, opening lines of communication, mutual understanding and respect. Improved renumeration could incentivize nurses to remain in Jamaica, leading to fewer *imbalances/inequities* in the nursing workforce. *Policy* changes allowing nurses an expanded scope of practice have the potential to positively influence nurse retention. For instance, granting community-based palliative care practice and opioid-based pain management privileges could entice nurses to remain in the country. A literature search for articles having to do with migration effects on palliative care integration efforts outside the Caribbean revealed a paucity of evidence. The only article identified described Israel’s introduction of advanced practice nurses with specialty palliative care training to counter physician workforce shortages and recommendations to rapidly expand the advanced practice nursing role to improve healthcare access and bolster existing resources creating a rich pool of health professionals [64]. Tapping into the pool of second generation migrants with nurse training who have the potential to improve the social and economic picture in Jamaica and the wider Caribbean is another strategy that should be further investigated through policy initiatives [63]. The IOM UN report discussed the potential of highly educated Jamaican diaspora to contribute to strengthening Jamaica’s social and economic picture, a tactic that has potential to boost palliative care development [55]. Tailored mitigation strategies to reduce nurse migration from Jamaica are profoundly important when considering palliative care integration and the key roles that nurses can play. In the absence of drastic improvements to *educational* offerings and restrictive *policies* related to nurses, out-migration of nurses will most certainly hamper palliative care integration efforts in Jamaica.

Although counter-intuitive, it is important to consider avenues in which nurse migration may benefit palliative care integration in Jamaica. Several identified themes align with the *Model of Sustainability in Global Nursing* [41]. According to the model, fostering collaborative, cross-cultural *partnerships* that focus on advancing education, leadership, policy advocacy, and interprofessional collaborations, incentivizes nurses to expand their knowledge and skills, which leads to empowerment and sustained outcomes, such as reduced out-migration. International and interprofessional *partnerships* promote sustainable health system improvements and should be incorporated in efforts to integrate palliative care in Jamaica [41, 65, 66]. These findings are in support of *partnerships* pursued through the PAHO/WHOCCs such as the UAB-UWI collaboration [42, 43]). This type of reciprocal partnership leads to positive sustainable changes, including opportunities to enhance palliative care training and service delivery in both Jamaica and the USA. Similarly, the World Bank report recommends strengthening partnerships to enhance and broaden the role of nurse tutors in Jamaica who are needed to effectively educate and train competent nurses capable of delivering quality palliative care [54]. Given the unknown effect of the COVID-19 pandemic on out-migration, capitalizing on existing partnerships and fostering new cross-border collaborations should become a priority.

### Future directions and impact

Despite an array of mitigation strategies to counteract nurse migration, evidence on their effectiveness is lacking. The four emerged themes*, globalization*, *recruitment*, *imbalance/ inequities*, and *mitigation strategies* must be considered in efforts to integrate palliative care in Jamaica. Additionally, the impact of collaborative partnerships informed by the *WHO PHM* and the *Model of Sustainability in Global Nursing* should be further explored. The hypotheses generated from this review can guide future scientific inquiry:

1. Bolstering palliative care education and training opportunities will incentivize nurses to remain in Jamaica and contribute to palliative care integration efforts.
2. Collaborative partnerships in palliative care education between Jamaica and wealthier countries will lead to sustained retention of nurses in Jamaica.
3. Policies that reward nurses for advanced knowledge and skills in palliative care will incentivize nurses and mitigate out-migration.

The impact of the recent COVID-19 pandemic must be factored into strategies for successfully palliative care integration in Jamaica. Suggestions in the International Council of Nurses (ICN) report, *COVID-19 and the International Supply of Nurses* [67], align with themes identified in this critical review including *education* and migration *policy*. The report highlights Jamaica as a particularly vulnerable country to out-migration of nurses. Given the country’s limited domestic training capacity, the impact of COVID-19 on the nursing workforce such as burnout, absenteeism, illness, and death is further magnified [67]. Economic disruption and long-term financial problems are expected to escalate and further exacerbate out-migration.

*Cancer Care in the Commonwealth Caribbean in COVID times* [68] suggests a need to reframe and re-emphasize priorities for cancer care due to the pandemic’s effects, which inherently creates a greater need for palliative care. This aligns with a focus on *education* to increase cancer and palliative care capacity [68]. Models of care that incorporate task-shifting (i.e., from physicians to nurses and from nurses to less-skilled workers) through *education,* and development of community-based services to accommodate social distancing and barriers to seeking care must be considered. An example of a *strategy* through *partnerships* included the use of telehealth with support from high-resource countries [68] and incorporating nurse tutors in Jamaica through online course experiences [54]. A focus on filtering more nurses into primary care settings through policy initiatives may also bolster community palliative care capacity in Jamaica [54]. Mitigating strategies coupled with travel restrictions that quell migration, may foster improved nurse-retention at least for the near future.

### Strengths and limitations

The investigators’ expertise in methodology as well as in the substantive content areas of palliative care, nurse migration, and global nursing is a main strong point of this critical review. Similarly to Rolle-Sands et al. [24], we explored empirical evidence pertaining to the effects of nurse migration on outcomes, including the experiences of those who remain in Jamaica and the outcomes of those they care for. However, we specifically focused on how these outcomes may impact palliative care advancement in Jamaica. Although there was relative paucity of robust studies, sufficient descriptive information and experiences were identified to formulate working hypotheses for future research. An attempt to identify additional resources specific to migration effects on palliative care integration outside of the Caribbean yielded little information. One article highlighted expansion of palliative-trained advanced practice nurses to fill physician workforce gaps in Israel; a strategy that may be useful in the Jamaican context [64].

## Conclusions

This critical review offers a comprehensive overview of the effects of nurse migration on palliative care integration in Jamaica. Emerged themes included: (a) *globalization* creating opportunities for migration, (b) *recruitment* of skilled professionals from CARICOM, (c) *imbalance and inequities* resulting from migration, and (d) *mitigation reduction strategies*. To fully contribute to palliative care integration, nurses require opportunities for *education* as well as *policy* support. Without significant *policy* change, such as expanding the role and capacity of Jamaican nurses, increasing salaries and offering leadership opportunities, nurses will continue to migrate. Continued loss of Jamaica’s skilled nursing workforce will negatively impact palliative care integration efforts and is a barrier to meeting the sixty-seventh World Health Assembly recommendations for strengthening palliative care [69]. Formulated hypotheses from this review should guide future investigation of the effects of nurse migration on integration of palliative care in Jamaica. Novel mitigating strategies, and opportunities for enhanced nurse education such as investigating the role of advanced practice nurses with specialty palliative care training, should be considered within Jamaica’s healthcare system.

## Data Availability

Data sharing is not applicable to this article as no datasets were generated or analyzed during the current study.

## Abbreviations

CARICOM: Caribbean Community and Common Marketplace
CINAHL: Cumulative Index to Nursing and Allied Health Literature
GATS: General Agreement on Trade and Services
ICN: International Council of Nurses
MMP: Managed Migration Program
PAIS: Public Affairs International Service
PHM: Public Health Model
PRISMA: Preferred Reporting Items for Systematic Reviews
USA.: United States of America
WHO: World Health Organization
